# Long noncoding RNA profiling revealed differentially expressed lncRNAs associated with disease activity in PBMCs from patients with rheumatoid arthritis

**DOI:** 10.1371/journal.pone.0186795

**Published:** 2017-11-15

**Authors:** Min Yuan, Shujun Wang, Lijie Yu, Bo Qu, Liming Xu, Lining Liu, Huanxia Sun, Chunxian Li, Yanjun Shi, Huaxiang Liu

**Affiliations:** 1 Department of Rheumatology, Shandong University Qilu Hospital, Jinan, China; 2 Department of Rheumatology, Liaocheng People’s Hospital, Liaocheng, China; 3 Department of Rheumatology, Central Hospital of Zibo, Zibo, China; 4 Department of Rheumatology, Dong’e People’s Hospital, Liaocheng, China; 5 Shanghai Institute of Rheumatology, Department of Rheumatology, Renji Hospital, School of Medicine, Shanghai Jiao Tong University, Shanghai, China; Kunming University of Science and Technology, CHINA

## Abstract

Long noncoding RNAs (lncRNAs) have recently emerged as important biological regulators, and the aberrant expression of lncRNAs has been reported in numerous diseases. However, the expression of lncRNAs in peripheral blood mononuclear cells (PBMCs) in rheumatoid arthritis (RA) has not been well documented. We applied a microarray analysis to profile the lncRNA and mRNA expression in 3 pairs of samples. Each sample was mixed with equivalent PBMCs from 9 female RA patients and 9 corresponding healthy controls, and the data were validated via qPCR using another cohort that comprised 36 RA patients and 24 healthy controls. A bioinformatic analysis was performed to investigate the potential functions of differentially expressed genes. Overall, 2,099 lncRNAs and 2,307 mRNAs were differentially expressed between the RA patients and healthy controls. The bioinformatic analysis indicated that the differentially expressed lncRNAs regulated the abnormally expressed mRNAs, which were involved in the pathogenesis of RA through several different pathways. The qPCR results showed that the expression levels of ENST00000456270 and NR_002838 were significantly increased in the RA patients, whereas the expression levels of NR_026812 and uc001zwf.1 were significantly decreased. Furthermore, the expression level of ENST00000456270 was strongly associated with the serum levels of IL-6 and TNF-a and the Simplified Disease Activity Index (SDAI) of the RA patients. Our data provided comprehensive evidence regarding the differential expression of lncRNAs in PBMCs of RA patients, which shed light on the understanding of the molecular mechanisms of lncRNAs in the pathogenesis of RA.

## Introduction

Rheumatoid arthritis (RA) is the most common form of inflammatory and destructive arthritis and affects 0.5% to 1% of the general population worldwide. As a chronic autoimmune disease that predominately occurs in women, the ratio of female-to-male RA patients is 3:1. Although the cause and pathogenesis of RA are complicated and multifaceted, and the precise mechanisms are not fully understood, the sustained inflammatory process and immune disorders caused by imbalance of regulatory mediators are well accepted[1, 2]. As a complicated disease, numerous cell types, including macrophages, T cells, B cells, neutrophils, mast cells, and dendritic cells, are involved in the pathogenesis of RA.

In recent years, lncRNAs have begun to receive substantial attention in the molecular biology field. Increasing evidence indicates that lncRNAs, as an versatile molecules that interact with RNA, DNA, or proteins to promote or restrain the expression of protein-coding genes, are involved in diverse biological processes, including cell proliferation, differentiation, apoptosis, and development[3–5]. Moreover, in the field of immunology, various studies have indicated that lncRNAs not only are emerging as important regulators of inflammatory gene expression in innate immune system[6, 7], but also play a crucial role in directing the development and apoptosis of diverse immune cells and controlling the dynamic transcriptional programs that are hallmarks of immune cells activation or differentiation in adaptive immune system[8–11]. LncRNAs form regulatory complexes that coordinate the development of immune cell lineages and control the gene expression programs in these cells[12]. The importance of lncRNAs is uncovered by their newly identified roles in several autoimmune diseases, including systemic lupus erythematosus (SLE), rheumatoid arthritis (RA), and primary Sjögren’s syndrome (pSS)[13–15]. Nevertheless, knowledge of lncRNAs in RA PBMCs, particularly in female patients, is lacking. In this study, we examined the lncRNA expression profile in female RA patients using microarray analysis to investigate the potential roles of lncRNAs in the pathogenesis of RA, assess the relationship between differentially expressed lncRNAs and clinical data, and explore novel biomarkers used in diagnosis, disease monitoring and prognosis.

## Methods

### Patients and healthy controls

Ethical approval for this study was provided by the Research Ethics Committee of Liaocheng People’s Hospital (registration number: 2016072). Between July 2016 and December 2016, patients with RA were recruited from the Department of Rheumatology of Liaocheng People’s Hospital. All patients with RA were diagnosed according to the ACR/European League Against Rheumatism 2010 classification criteria for RA[16]. All participants provided informed consent prior to enrollment. Healthy controls were enrolled from volunteers of the health examination center of Liaocheng People’s Hospital and had no history of autoimmune disease or smoking. The patients with RA and the healthy controls did not significantly differ with respect to mean age or sex distribution. Individuals with a current or recent infection were excluded from the study. In the microarray analysis, 3 pairs of samples from 27 female RA patients [mean age (± SD) 49.02 yrs (± 10.63)] and 27 corresponding healthy controls [mean age (± SD) 51.04 yrs (± 9.37)] were analyzed. Each sample comprised equivalent PBMCs from 9 female RA patients and 9 corresponding healthy controls. The validation cohort consisted of 36 RA patients [61.11% women, mean age (± SD) 50.34 yrs (± 9.09), mean disease course11.32 yrs] and 24 healthy controls [58.33% women, mean age (± SD) 48.55 yrs (± 8.63)]. The mean serum levels of IL-6 and TNF-α of the RA patients in the validation cohort were 63.12 pg/ml and 646.36 pg/ml, respectively. For each patient in the validation cohort, the disease severity was assessed with the simplified disease activity index (SDAI). The SDAI is calculated using the numerical sum of the following five outcome parameters: tender and swollen joint counts (based on a 28-joint assessment), patient and physician global assessment of disease activity [visual analogue scale (VAS) 0–10 cm] and level of C-reactive protein (mg/dl, normal <0.8 mg/dl)[17].

### Blood samples and RNA isolation

Peripheral blood samples (10 ml) were obtained from each subject. The samples were collected in ethylene diamine tetraacetic acid (EDTA) tubes from July 2016 to December 2016. PBMCs were isolated using Ficoll density gradient centrifugation and were counted with a blood cell analyzer (Sysmex, Kobe, Japan). Total RNA was subsequently extracted from the PBMCs using TRIzol (Invitrogen, Carlsbad, CA, USA). The RNA integrity was evaluated with standard denaturing agarose gel electrophoresis, and the RNA concentrations were measured using a NanoDrop1000 spectrophotometer (NanoDrop Technologies, Wilmington, DE, USA) with a 260 nm/280 nm ratio greater than 1.8.Approximately 1 μg of total RNA was reverse-transcribed into cDNA using a PrimeScript RT reagent kit (TaKaRa, Dalian, China). All RNA and cDNA samples were stored at -70°C prior to use.

### Microarray analysis

Three pairs of samples were prepared for lncRNA microarray analysis using an Arraystar Human lncRNA Microarray V4.0 (Arraystar, Rockville, MD, USA). Approximately 40,173 lncRNAs and 20,730 coding transcripts can be detected by this third-generation lncRNA microarray. Sample labeling and array hybridization were performed according to the Agilent One-Color Microarray-Based Gene Expression Analysis protocol (Agilent Technologies, Santa Clara, CA, USA) with minor modifications. All microarray work was performed by Kangcheng Bio-Tec (Shanghai, China). Agilent Feature Extraction software (version 11.0.1.1) was used to analyze the acquired array images. Quantile normalization and subsequent data processing were performed using the GeneSpring GX v12.1 software package (Agilent Technologies, Santa Clara, CA, USA). Following quantile normalization of the raw data, lncRNAs and mRNAs for which at least 3 of 6 samples have flags in Present or Marginal (“All Targets Value”) were selected for further data analysis. Differentially expressed lncRNAs and mRNAs between the two groups were identified through *P*< 0.05 and a fold-change > 2.0. Hierarchical clustering and combined analysis were performed using in-house scripts.

### Bioinformatic analysis

Pathway analysis and Gene Ontology (GO) analysis were applied to explore the potential roles that the differentially expressed mRNAs played in biological pathways or GO terms, including the following three categories: biological process, cellular component, and molecular function. A co-expression analysis was performed by associating the expression profiles of DE-lncRNAs with DE-mRNAs. This association was based on the information of the lncRNA-associated coding regions, which was supplied by the microarray analysis.

### Real-time quantitative polymerase chain reaction

Complementary DNA (cDNA) was amplified via RT-PCR using SYBR Green (TaKaRa, Dalian, China). Primers were designed and synthesized by Generay Biotech (Shanghai, China). The thermocycling protocol included the following steps: denaturation for 30 s at 95°C and then 40 cycles of 95°C for 5 s and 60°C for 30-60s. After amplification, a melting curve analysis was performed at 95°C for 15 s and 60°C for 1 min to monitor primer dimers or nonspecific product formation. GAPDH mRNA was used as an endogenous control to normalize the lncRNA expression levels using the 2^-ΔΔCT^ method. All reactions were performed in triplicate. The primer sequences used for RT-PCR were as follows: NR_002838, 5’-GAG CCG ATC TTA CAA CCT C-3’(forward) and 5’-TGG ATC TCT ACT AGC CAC A-3’(reverse);uc001zwf.1, 5’-ATT GGC ATA ATA CAT TCC C-3’(forward) and 5’-TGG CTA TAA AAG GTA AAT GCA A-3’(reverse); ENST00000456270, 5’-AGC CAG CAT AAC AAG AGT TCC-3’(forward) and 5’-ACA CGT TCC AGT AGA ATG CC-3’(reverse); NR_026812, 5’-ATT TAT CGA ACC TCA CGG AGC-3’(forward) and 5’-TCT TTA GCT TCT GTA GTT CGG-3’(reverse);ENST00000412896, 5’-AGC CAG CAT AAC AAG AGT TCC-3’(forward) and 5’-ACA CGT TCC AGT AGA ATG CC-3’(reverse); ENST00000566394, 5’-CTA GAG TCC ATG TTG GCC TT-3’(forward) and 5’-TGC AGG TCT TCT ATG ACG TT-3’(reverse); and GAPDH, 5’-TCT GAC TTC AAC AGC GAC ACC-3’(forward) and 5’-TGT TGC TGT AGC CAA ATT CGT-3’(reverse).

### Statistical analysis

GraphPad Prism 5.0 (GraphPad Software, La Jolla, CA, USA) was used to analyze the data. The nonparametric Mann-Whitney test was used to compare the gene expression between two groups. The correlations between the expression levels of lncRNAs and the clinical characteristics were analyzed using the Spearman’s rank correlation coefficient test. *P*-values (two-tailed) <0.05 were considered statistically significant.

## Results

### Characteristics of differentially expressed lncRNAs and mRNAs in the PBMCs of RA patients

**Fig 1:**
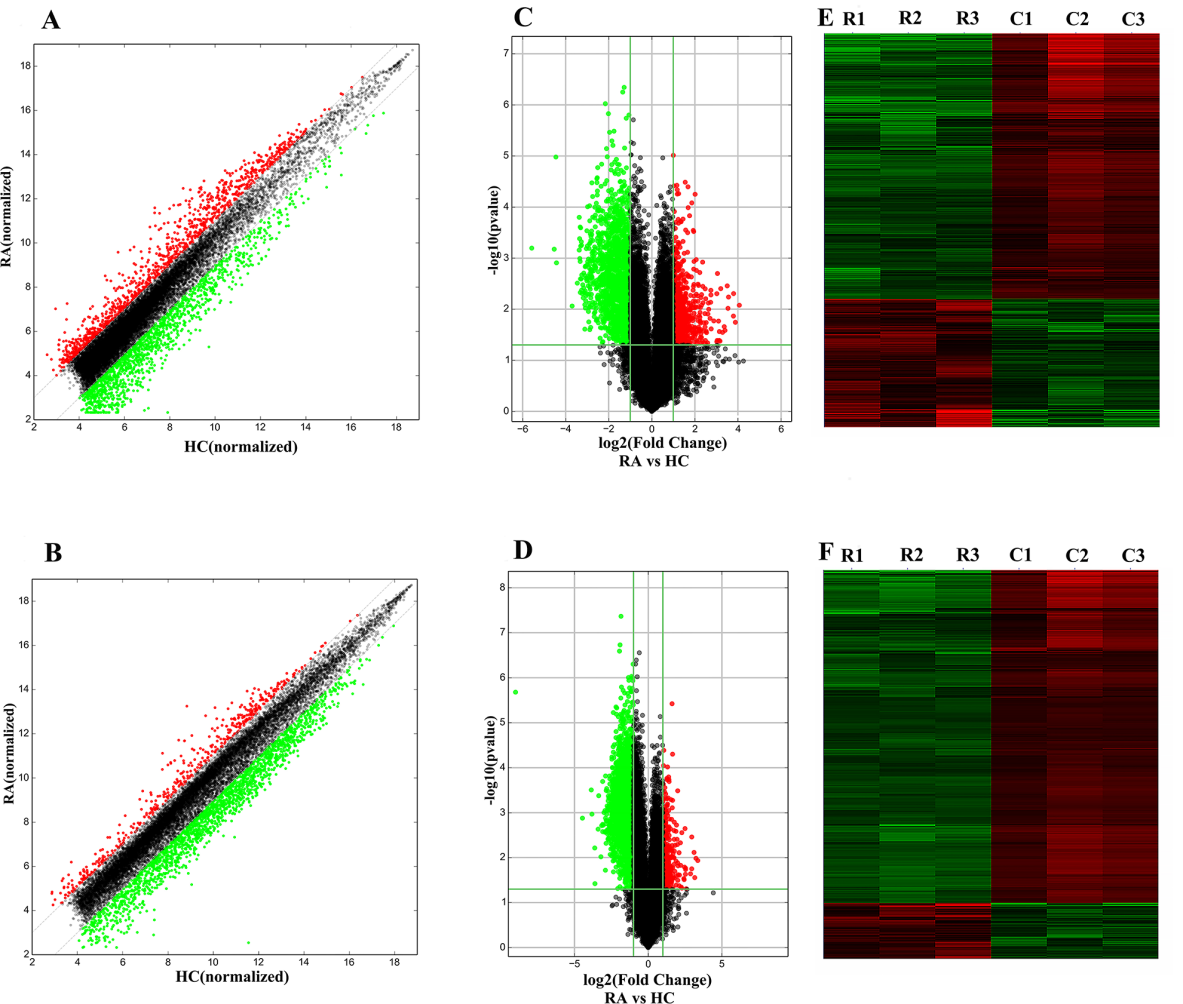
Expression profiles of lncRNAs(A/C/E) and mRNAs(B/D/F) in female RA patients. (A and B) Scatter-plot of differences in the lncRNA and mRNA expression. The X and Y axes are the mean normalized signal values (log2 scaled). The gray lines are 2-fold change lines. (C and D) Volcano plot of differentially expressed lncRNAs and mRNAs. The horizontal green line represents a P-value of 0.05, and vertical green lines represent 2.0-fold changes up and down. X axes are the fold change values (log2 scaled), and Y axes are the P-values (log10 scaled). (E) and (F) Hierarchical clustering analysis of lncRNAs and mRNAs with expression changes greater than two-fold and *P* value <0.05. RA patient group: R1, R2, R3; Healthy control group: C1, C2, C3. Red and green colors represent up- and downregulated genes, respectively.

**Fig 2:**
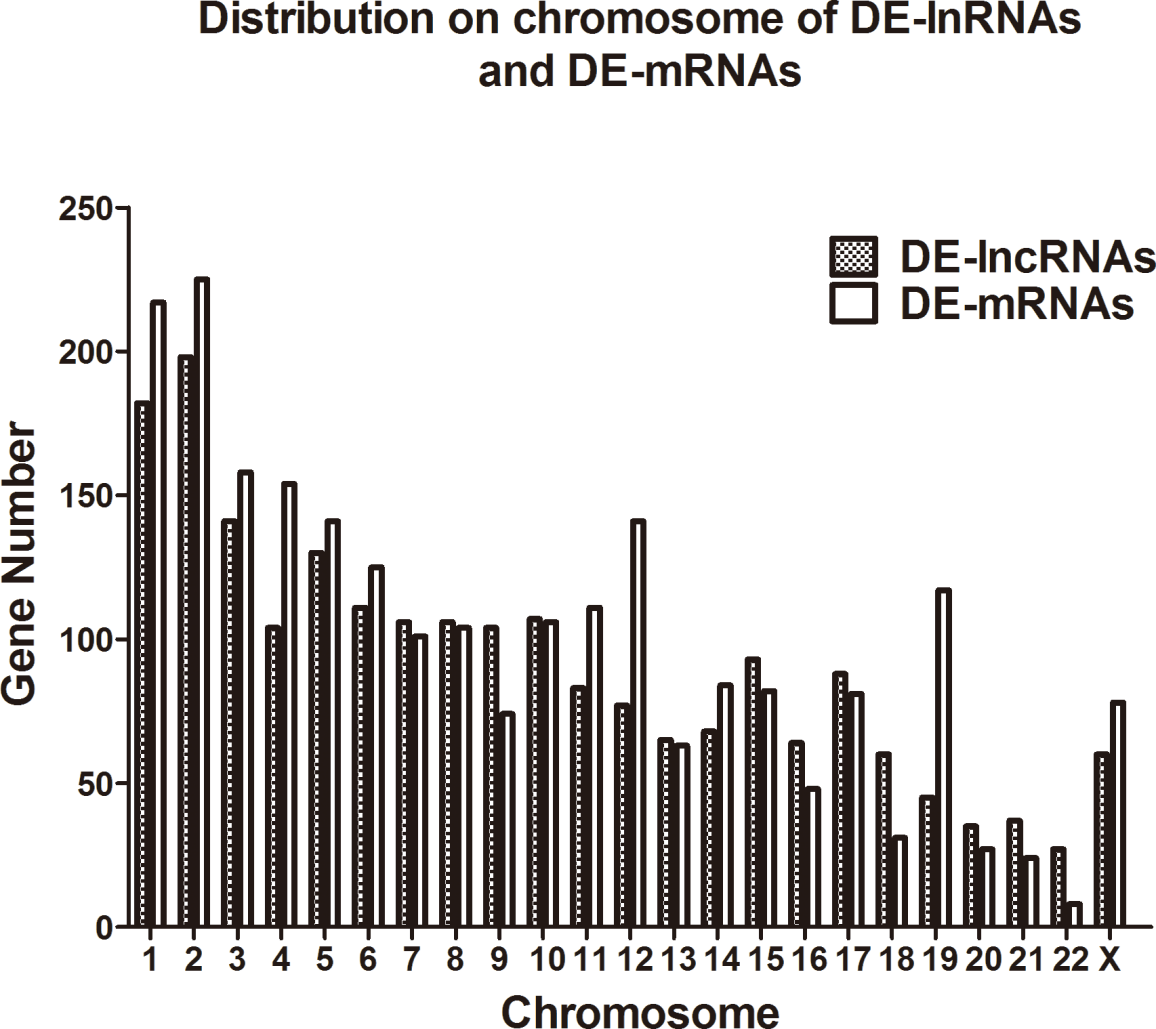
Distribution of differentially expressed lncRNAs and mRNAs on chromosomess.

The PBMC samples from 27 female RA patients and 27 healthy controls were randomly divided into three pairs with each pair containing age matched samples from RA patients or healthy controls. Based on the cutoff criteria of a fold change >2.0 and a P value<0.05, 2,099 lncRNAs and 2,307 mRNAs were significantly differentially expressed in all three pairs. Among the 2,099 differentially expressed lncRNAs (DE-lncRNAs), 683 were upregulated, and 1,416 were downregulated. Among the 2,307 differentially expressed mRNAs (DE-mRNAs), 331 were upregulated and 1,976 were downregulated. The differences in the lncRNA and mRNA expression are presented as scatter plots (Fig 1A and 1B) and evaluated by volcano plot filtering (Fig 1C and 1D). The hierarchical clustering analysis (Fig 1E and 1F) showed distinct expression signatures of both lncRNAs and mRNAs. We analyzed the distribution of DE-lncRNAs and DE-mRNAs on chromosomes. The results indicated there was no distinct enrichment of DE-lncRNAs or DE-mRNAs on specific chromosomes (Fig 2).

**Fig 3:**
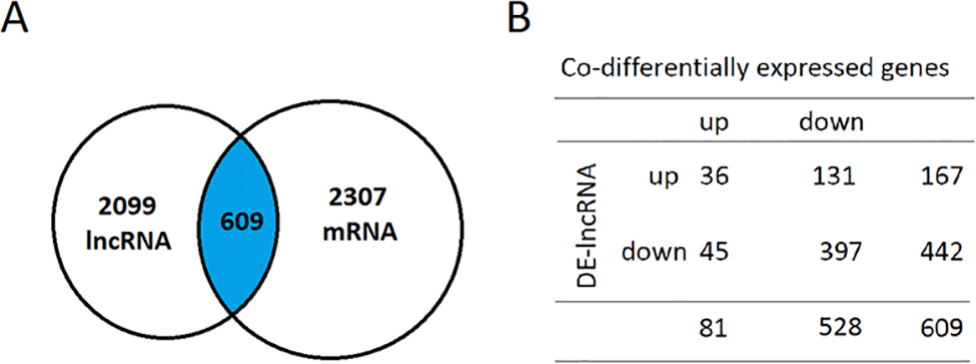
Expression relationship of DE-lncRNA-associated coding genes (distance<100kb) and DE-mRNAs in RA. (A) The crossover of DE-lncRNA-associated coding genes with DE-mRNAs. (B) The table lists the expression correlation of DE-lncRNAs and their neighboring coding genes.

Several studies have reported that lncRNAs control the expression of genes that are positioned in the vicinity of their genomic location[5, 18, 19]. Based on this theory, we investigated whether the mRNAs transcribed from the loci close (<100kb) to the coding region of the 2,099 differentially expressed DE-lncRNAs were also differentially expressed in our data. We found 609 out of 2,099 DE-lncRNAs had their neighboring mRNAs also differentially expressed in our data (Fig 3A). Among these 609 DE-lncRNAs, 338 were localized upstream of the neighboring DE-mRNA genes, whereas 271 were localized downstream. Further analysis showed 433 DE-lncRNAs were expressed in the same direction with their neighboring mRNA genes, whereas 176 DE-lncRNAs showed different expression directions(Fig 3B). We also determined that there may be more than one DE-mRNA located around one DE-lncRNA; moreover, some DE-mRNAs have multiple associated adjacent DE-lncRNAs, which indicated complicated regulatory mechanisms by DE-lncRNAs for the expression of DE-mRNAs. To improve understanding the regulatory roles of lncRNA and its possible enhancer roles in neighboring genes[20], we focused on one of subgroup of DE-lncRNAs, differentially expressed long intergenic noncoding RNAs(DE-lincRNA) and their differentially expressed neighboring protein-coding genes (distance < 300 kb) in detail. There are 435 differentially regulated lincRNAs, of which 131 lincRNAs have two or more adjacent protein-coding genes, and the total number of adjacent protein-coding genes is 303. Of these adjacent protein-coding genes, 159 were located upstream of lincRNAs, and 144 were located downstream. It was noteworthy that of the 131 DE-lincRNAs with their 303 adjacent mRNA pairs, 230 pairs exhibited a similar expression direction (upregulated or downregulated), whereas 73 pairs exhibited different expression directions. Furthermore, we determined that there were 500 differentially expressed neighboring protein-coding genes, of which 76 neighboring protein-coding genes had two or more adjacent lincRNAs, and the total number of adjacent lincRNAs was 183. Of these 183 lincRNAs, 81 lincRNAs were located upstream of protein-coding genes, and 102 were located downstream. One hundred twenty-five lincRNAs exhibited a similar expression direction with their adjacent protein coding genes, whereas 58 lincRNAs exhibited different expression directions (Table 1).The detailed relationship regarding the genomic location between these lincRNAs and adjacent protein-coding genes is presented in S1 Table.

**Table 1:** Analysis of DE-lincRNAs and neighboring protein-coding genes (distance < 300 kb).

	Neighboring protein-coding genes (303)	lincRNAs (183)
**Upstream**	159	102
**Downstream**	144	81
**Same expression direction**	230	125
**Different expression direction**	73	58

Upstream: lincRNAs located upstream of neighboring protein-coding genes; downstream: lincRNAs located downstream of neighboring protein-coding genes; same expression direction: both lincRNAs and adjacent coding genes are either upregulated or downregulated; different expression direction: lincRNAs or adjacent coding genes are upregulated, whereas the others are downregulated.

### Pathway and GO analyses indicated potential pathways regulated by differential lncRNAs in RA patients

**Fig 4:**
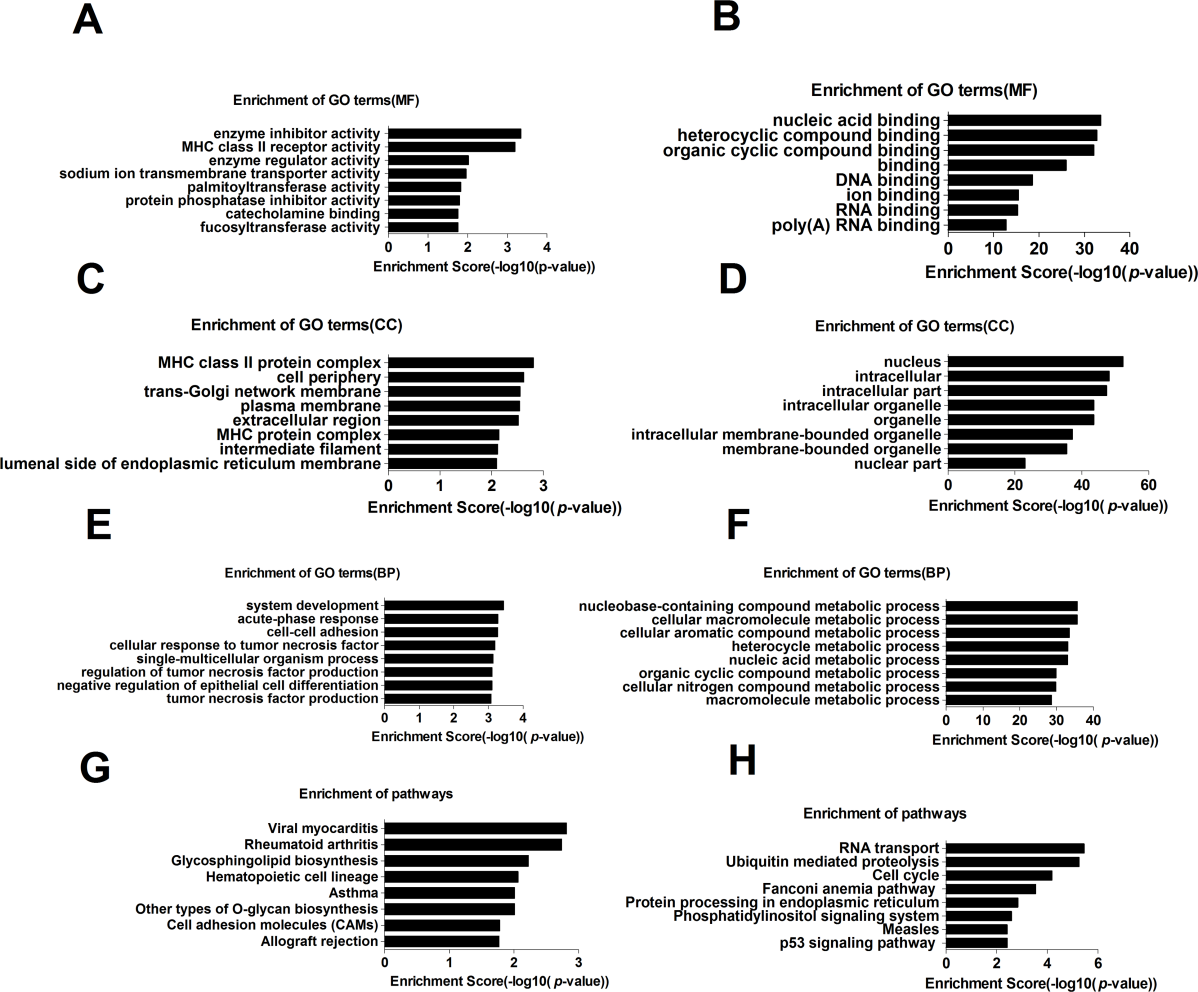
Enrichment analysis of GO terms and pathways for differentially expressed mRNAs. The top 8 GO analyses (which consisted of a cellular component (CC), molecular function (MF), and biological process (BP)) (A-F) and top 8 pathways (G,H) that exhibited significant differences between RA and HC are listed (left and right panels show the upregulated and downregulated coding genes, respectively).

Pathway and GO analyses were performed to explore the potential roles that the aberrantly expressed mRNAs may play in RA. The GO analysis indicated that the most significantly enriched molecular functions of upregulated mRNAs in the PBMCs of RA were enzyme inhibitor activity, MHC class II receptor activity and enzyme regulator activity (Fig 4A). The downregulated genes were mainly involved in nucleic acid binding, heterocyclic compound binding and organic cyclic compound binding (Fig 4B).The most significantly enriched cellular components of the upregulated mRNAs were MHC class II protein complex, cell periphery and trans-Golgi network membrane(Fig 4C). The significantly enriched cellular components of the downregulated mRNAs were the nucleus and intracellular and intracellular parts (Fig 4D). The most significantly enriched biological processes of the upregulated mRNAs were system development, acute-phase response and cell-cell adhesion (Fig 4E), whereas the downregulated mRNAs were involved in a series of nucleus and cellular compound metabolic processes (Fig 4F). The results of the pathway analysis indicated that the upregulated mRNAs in the PBMCs of RA patients were significantly involved in viral myocarditis, rheumatoid arthritis, glycosphingolipid biosynthesis, and cell adhesion molecules (CAMs) (Fig 4G). The downregulated mRNAs were significantly involved in the cell cycle, ubiquitin-mediated proteolysis and RNA transport (Fig 4H).

### qPCR validated 4 differentially expressed lncRNAs in the PBMCs of RA patients

**Fig 5:**
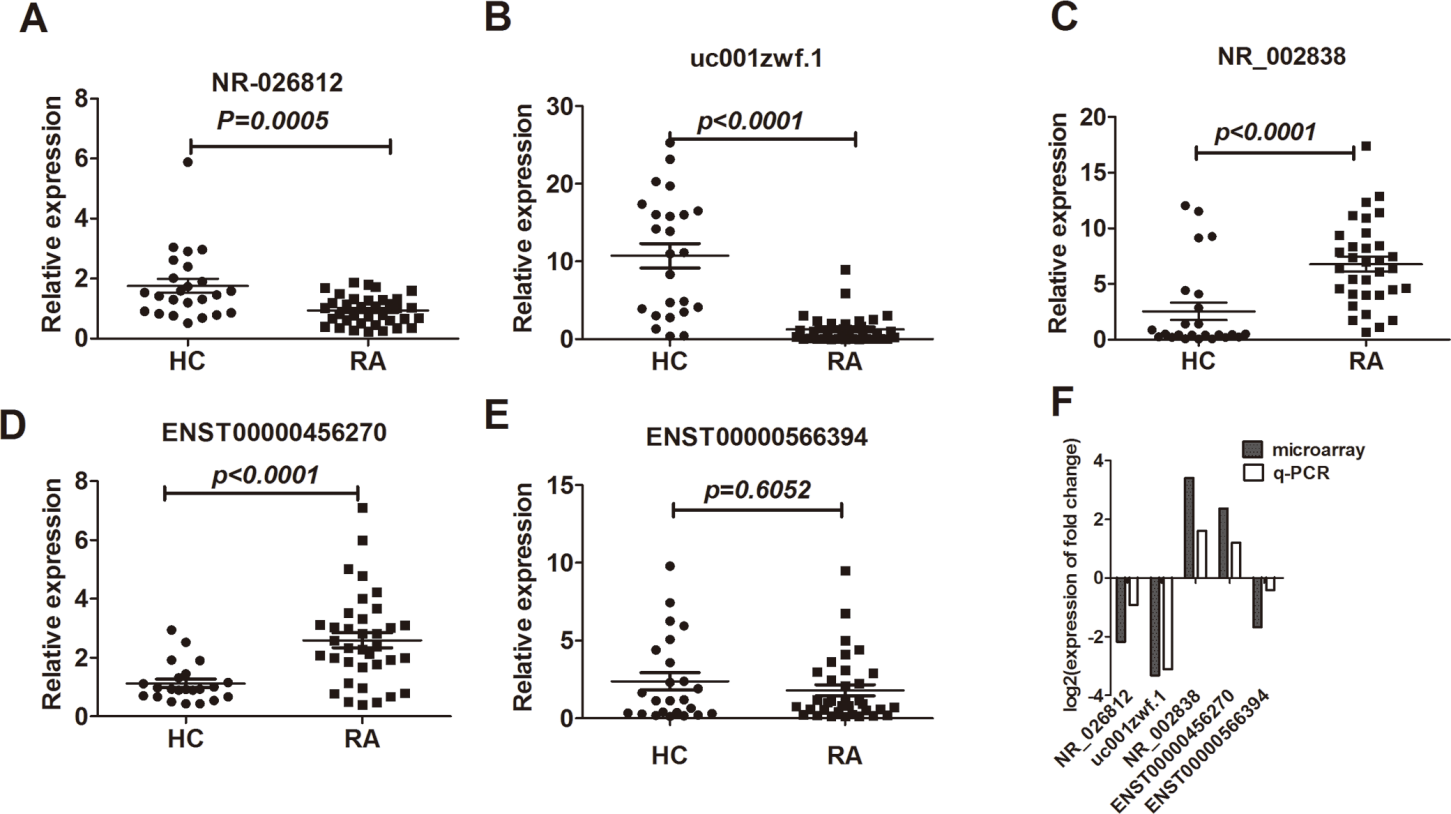
Relative expression (qPCR) of lncRNAs from RA patients and HCs. (A–E) The expression levels of five selected lncRNAs in 36 RA patients and 24 corresponding HCs. (A-D) The expression levels of four lncRNAs (NR_002838, uc001zwf.1, ENST00000456270, and NR_026812) in RA patients and HCs were significantly different. (E) The difference in lncRNA ENST00000566394 was not significant. (F) Comparison of the expression of five validated lncRNAs in microarray and qPCR results. A negative number indicates that lncRNAs were downregulated, and a positive number indicates the upregulation of lncRNAs. The heights of the columns in the chart represent the mean expression value of the log2 fold changes (RA/HC) for each of the five validated lncRNAs in the microarray and RT-qPCR data.

**Fig 6:**
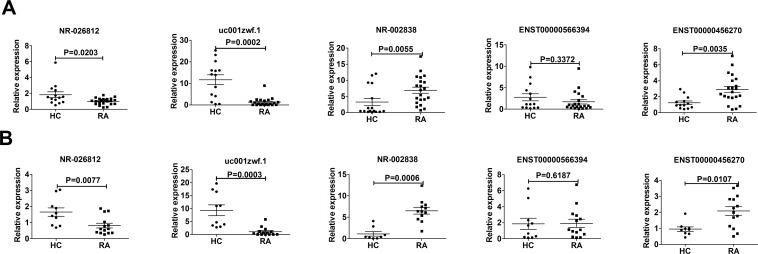
Relative expression of lncRNAs from female and male RA patients and in HCs. (A) The expression levels of five selected lncRNAs in 22 RA female patients and 14 corresponding HCs. (B) The expression levels of five selected lncRNAs in 14 RA male patients and 10 corresponding HCs. The expression levels of four lncRNAs (NR_002838, uc001zwf.1, ENST00000456270, and NR_026812) in RA patients and HCs were significantly different, whereas the difference in lncRNA ENST00000566394 was not significant.

To validate the microarray data, 6 candidate lncRNAs were selected for validation in the validation cohort, which consisted of 36 RA patients and 24 healthy controls. We selected the lncRNAs based on the fold changes in expression (fold changes>3, *P*<0.05), the length of each lncRNA (length<2500 bp), whether the lncRNAs have definite sequences, and whether the contexts of the genes are associated with RA. The qPCR results confirmed that ENST00000456270 and NR_002838 were increased in the RA PBMCs, whereas the expression levels of NR_026812, uc001zwf.1 and ENST00000566394 were decreased. The changes were statistically significant for 4 lncRNAs (Fig 5A–5D), and only the change of one lncRNA, ENST00000566394, was not statistically significant (p = 0.6052) (Fig 5E). All five aberrantly expressed lncRNAs showed the same trends in the qPCR and microarray analyses (Fig 5F). The expression level of ENST00000412896 was too low (CT>33) in the preliminary experiment to be analyzed by qPCR. Moreover, we further analyzed the expression of the five aberrantly expressed lncRNAs between the male and female patients, and the results were the same (Fig 6). Using the analysis of the raw value of the selected genes and standards that Wang *et al*[21] and Shi *et al*[15] adopted, the raw expression intensity of the probes of the selected genes in each sample must be greater than 100. These results indicated that the expression levels of ENST00000456270, NR_002838, NR_026812 and uc001zwf.1 were significantly dysregulated in RA; therefore, we selected these four lncRNAs for further analysis.

### Expression of lncRNAs associated with RA clinical features

**Fig 7:**
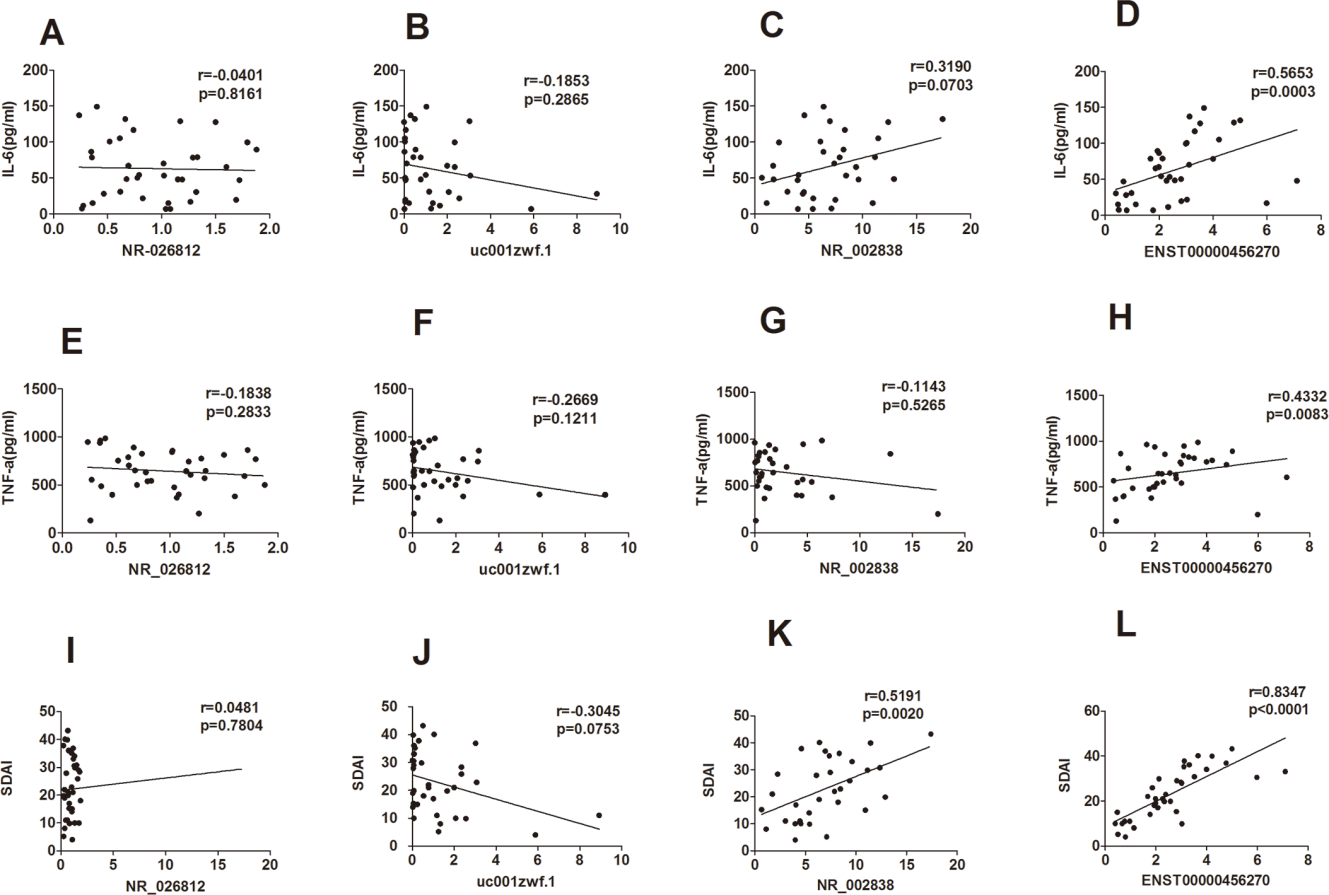
Correlations between differentially expressed lncRNAs and clinical characteristics in RA. (A-H) The expression level of ENST00000456270 was significantly positively correlated with serum levels of IL-6 and TNF-a; (I-L) the expression levels of NR_002838 and ENST00000456270 were significantly correlated with the SDAI.

We analyzed the correlation between the expression levels of DE-lncRNAs and clinical characteristics. The results showed that the expression levels of NR_002838 and ENST00000456270 were strongly correlated with the Simplified Disease Activity Index (SDAI) (r = 0.5191, p = 0.0020 and r = 0.8347,p<0.0001, respectively), and the expression level of ENST00000456270 was significantly associated with the serum levels of IL-6 (r = 0.5653, *P* = 0.0003) and TNF-α (r = 0.4332, *P* = 0.0083); NR_026812 and uc001zwf.1 had no significant correlation with IL-6, TNF-α and SDAI. Furthermore, the correlation of NR_002838 with IL-6 and TNF-α was not significant (Fig 7).

## Discussion

Following the development of high-throughput genetic sequencing technology, an increasing number of lncRNAs have been recognized. As a novel class of non-coding RNAs with a length longer than 200 nucleotides, lncRNAs are beginning to be understood. Although lncRNAs have no protein coding potential, these molecules may affect nearly all stages of gene regulation, including chromatin marks, signaling pathways, and mRNA processing, via modular domains that bridge the DNA, RNA, and protein interactions[22]. The biological functions of lncRNAs are constantly being explored, particularly in the field of autoimmune diseases, and additional studies are being performed [13, 23–25]. Recent progress has been made in the field of RA. Yang *et al* reported that lncRNA-NR024118, which may be upregulated by Shikoninin, the CAIA mouse model of RA, has the ability to inhibit the inflammatory response[26]. lncRNA-p21 has a low expression in RA, and methotrexate (MTX) can restore lncRNA-p21 levels to normal in vivo, which indicates that lncRNA-p21 may play an important role in the anti-inflammatory properties of MTX[27]. LOC100506036 may contribute to the inflammatory responses in RA by regulating several genes, including SMPD1 and NFAT1[14]. Zhang *et al* explored the expression profile in the fibroblast-like synoviocytes of RA and identified differently expressed lncRNAs that may participate in the pathogenesis of RA[28]. However, few studies have focused on lncRNA expression profiles in PBMCs of RA patients. Our study identified differentially expressed lncRNAs in the PBMCs of female RA patients for the first time, as well as potential roles of DE-lncRNA in the pathogenesis of RA.

A high ratio (two thirds to three quarters) of RA patients are female. To avoid sex differences, we selected female patients as the study subjects in the microarray analysis to ensure the accuracy and representativeness of the results. Our results showed that the majority of DE-lncRNAs and DE-mRNAs are downregulated. The number of downregulated lncRNAs was 2-fold higher than the number of upregulated lncRNAs and the number of DE-mRNAs was 5.96-fold higher, which indicates that a deficiency of gene expression or increased RNA degradation may play an important role in the pathogenesis of RA; further research should be made to examine which mechanism is predominant. However, some differentially expressed lncRNAs identified by Zhang *et al*[28] in the RA FLSs were not as differentially expressed or detected in our microarray analysis, which may be a result of the cell specificity of lncRNAs[29]. In terms of the chromosome distribution of aberrantly expressed lncRNAs and mRNAs, our study indicated that DE-mRNAs and DE-lncRNAs were predominately distributed on chromosome 2 and that they were rarely distributed on chromosome 22. In the detailed analysis of DE-lincRNAs and differently expressed neighboring protein-coding genes, we determined one lincRNA had two or more (the most is six) neighboring protein-coding genes and a protein-coding gene associated with multiple aberrantly expressed lincRNAs. Moreover, we also discovered that approximately 70% of lincRNAs and adjacent coding genes exhibited the same expression direction, which may improve the knowledge of the mechanisms by which lincRNAs act. As described in several studies[30, 31], lincRNAs had highly conserved promoter regions that can recruit and bind key transcription factors. About a third of lincRNAs were found to associate with chromatin modifying complexes and modulate cellular pathways, which indicated that lincRNAs may regulate gene expression through a complex network. Although these positional and expressional relationships cannot prove that lincRNAs have a function in these adjacent protein-coding genes, they provide a hypothesis for targeted loss-of-function experiments.

DE-mRNAs were further analyzed by GO term enrichment and pathway enrichment analyses. The GO results indicated that the most significantly enriched molecular functions of unregulated mRNAs included enzyme inhibitor activity, MHC class II receptor activity, and enzyme regulator activity. The most significantly enriched cellular components of upregulated mRNAs included the MHC class II protein complex, cell periphery trans-Golgi network membrane, and plasma membrane. Genetic factors clearly play a role in the RA risk, severity, and progression. MHC class II is the most important genetic risk allele and accounts for approximately 40% of the genetic influence[1]. MHC class II transgenic mice, an important animal model of RA, have been widely employed in RA research[32]. A recent functional analysis indicated that MHC II may play a pivotal part in the production of autoantibodies in RA patients[33]. Positive regulation of cell proliferation, the acute-phase response, cell-cell adhesion, and the cellular response to tumor necrosis factor (TNF) were the most significantly enriched biological processes of the unregulated mRNAs. The pathway analysis results suggested that the increased mRNAs in the PBMCs of the RA patients were significantly involved in viral myocarditis, RA, glycosphingolipid biosynthesis, and cell adhesion molecules (CAMs); these results are consistent with the established mechanisms of RA, including virus infection [34, 35], autoimmunity[1], and material metabolism[36]. The significantly enriched molecular functions of decreasing mRNA showed dysfunction of the binding of RNA, DNA, and iron, whereas the enriched cellular components of downregulated mRNAs were mainly involved in the nucleus, intracellular organelles, and membrane-bounded organelles. The enriched biological processes demonstrated the deficiency of a series of metabolic processes, such as a nucleobase-containing compound metabolic process and a cellular molecule metabolic process. Moreover, the decreased mRNA affects the cell cycle, ubiquitin mediated proteolysis, and RNA transport. Low expression levels of these coding genes mainly affect the normal metabolism and function of molecules and cells, which supports the role of abnormally expressed coding genes in the pathogenesis of RA.

qPCR was performed to validate the lncRNA microarray results. In the microarray analysis, ENST00000456270, NR-026812, NR-002838 and uc001zwf.1 were differentially expressed, as indicated by the qPCR results. For the low expression of ENST00000412896 (CT>33), we did not analyze the difference between RA patients and healthy controls, further experiments such as subcellular fractionation method and/orRNA-seq are needed to test the low abundantlncRNAs[37]. Based on the qPCR results and the characteristics of the microarray data, the raw data of a candidate lncRNA must be greater than 100. The same results of the respective analysis of the five aberrantly expressed lncRNAs between the male and female patients indicated that our microarray results not only exclude the disturbance of sex but also serve as a representative to some extent.

RA is a common chronic inflammatory and destructive arthropathy. Both TNF-α and interleukin-6 (IL-6) play major roles in the pathogenesis of rheumatoid arthritis. The serum and synovial concentrations of both cytokines are high in patients with RA. As an important pro-inflammatory factor, TNF-α is an autocrine stimulator as well as a potent paracrine inducer of other inflammatory cytokines, including interleukin-1 (IL-1), IL-6 and interleukin-8 (IL-8). TNF-α not only stimulates fibroblasts to express CAMs that can increase the transportation of leukocytes into inflammatory sites but also increases osteoclast-mediated bone resorption [38, 39]. IL-6 is a pleiotropic pro-inflammatory cytokine involved in diverse biologic processes, such as inducing the final maturation of B cells into plasma cells, the activation of T cells, the induction of the acute-phase response, and the proliferation of synovial fibroblasts[2]. Moreover, the serum levels of IL-6 and TNF-α are important parts of the Multi-Biomarker Disease Activity (MBDA) score, and both factors reflect disease activity in RA, are predictive for radiographic progression, and indicate the risk of an RA flare after a drug reduction[40, 41]. The SDAI is a valid, sensitive assessment of disease activity and treatment response. It is easy to calculate and is thus a common tool for the clinical assessment of RA[17]. In the correlation analysis, we determined that only the expression level of ENST00000456270 was related to the serum levels of IL-6, TNF-α, and the SDAI, which indicates that ENST00000456270 may be a biomarker for assessing disease activity. ENST00000456270 is a 376-bp intronic antisense lncRNA transcript from the AC000111.6 gene located on chromosome 7. On the opposite strand of the same position of chromosome 7 is the coding gene CFTR, a membrane protein that belongs to the ABC transporter family; it functions as a chloride/anion channel in epithelial cells around the body and is responsible for cystic fibrosis (CF)[42]. Studies have shown that CFTR mutations in patients with RA appear to be an important marker of the risk of associated diffuse bronchiectasis (DB), which has been linked to a less favorable prognosis[43]. No aberrant expression of CFTR was identified in the current microarray analysis, and our subjects did not have a high rate of RA with diffuse bronchiectasis (DB), which indicates that the function of ENST00000456270 may not be related to CFTR; however, ENST00000456270 may be a biomarker for the diagnosis of RA and may play an important role in the pathogenesis of RA, which requires further research to validate. It would help to perform a prediction of ENST00000456270 targets and a functional analysis. Furthermore, it will be important to explore the expression level in other autoimmune diseases, such as SLE/pSS, to verify the definite role of ENST00000456270 in the assessment and diagnosis of RA.

In this study, we identified aberrant expression profiles of lncRNAs in female RA patients for the first time. Overall, 2,099 lncRNAs and 2,307 mRNAs were differentially expressed, and 6 lncRNAs were further validated by qPCR in 36 RA patients. An increasing level of ENST00000456270 was correlated with the SDAI and the serum IL-6 and TNF-αlevel in patients with RA. Our results improved our understanding of the molecular mechanisms of genes and lncRNAs in RA, and we identified one lncRNA, ENST00000456270, that may serve as a potential biomarker for the assessment and diagnosis of RA patients.

## Supporting information

S1 Table Positional relationship between lincRNAs and the adjacent protein-coding genes. (XLSX)
